# Endoscopic transnasal resection of the CP angle schwannoma

**DOI:** 10.3171/2020.4.FocusVid.19891

**Published:** 2020-04-01

**Authors:** Satoshi Kiyofuji, Masahiro Shin, Kenji Kondo, Tsukasa Koike, Taichi Kin, Nobuhito Saito

**Affiliations:** Department of Neurosurgery, University of Tokyo Hospital, Tokyo, Japan

**Keywords:** endoscopic surgery, lower cranial nerve schwannoma, three-dimensional fusion images, transnasal surgery, transsphenoid surgery, video

## Abstract

Cerebellopontine (CP) angle tumors are often resected via retrosigmoid craniotomy; however, sometimes cranial nerves (CNs) make their resection more complex. In such cases, the endoscopic transnasal approach can avoid such manipulations as delivering surgical instruments over CNs or peeling off CNs from the tumor, minimizing the risk of postoperative deficits. A 35-year-old man presented with a 37-mm cystic tumor in the right CP angle, and preoperative 3D fusion images revealed that multiple CNs (VII, VIII, and lower CNs) were running on the tumor posteriorly. The endoscopic transnasal approach enabled safe subtotal resection without causing neurological deficits, and the patient underwent stereotactic radiosurgery for the residual schwannoma.

The video can be found here: https://youtu.be/xKLwdDsLpWA.

## Transcript

This video demonstrates endoscopic transnasal resection of the CP angle schwannoma.

### 0:26 Case presentation

An otherwise healthy 35-year-old man presented with chronic dizziness for a few years. MRI obtained by a local ENT physician revealed a mass in the right CP angle. Neurologically, he was intact and did not have facial weakness, hearing loss, dysphagia, or dysarthria. His father had a surgery for cervical spine tumor, but details were not obtained.

### 0:50 Preoperative imaging studies

MRI with contrast demonstrated a 40-mm cystic tumor with an enhanced nodule in the right CP angle. The tumor approximated to the right jugular foramen.

### 1:01 3D fusion images

This is the anterior posterior view of the 3D fusion images constructed from multimodality imaging studies.^1^ The tumor was resided below the VII and VIII complex, lateral to the sixth nerve, and anterior to the lower cranial nerves. The enhanced nodule is demonstrated in purple, and this was sitting anterior to the tumor.

### 1:25 Simulation of surgery, retrosigmoid approach versus endoscopic transnasal approach

We discussed retrosigmoid approach versus endoscopic transnasal approach.^2^

In the retrosigmoid approach, most of the tumor is hidden by the cerebellum, and retraction of the cerebellum of a young patient deemed necessary. Furthermore, manipulation around the lower cranial nerves was inevitable to approach the enhanced nodule.

Contrarily, in endoscopic transnasal approach, we can approach directly to the enhanced nodule once we open the clivus, and manipulation of the lower cranial nerves is minimal. Thus, we selected endoscopic transnasal approach.

### 2:06 Incision to the nasal septum mucosa

First, we dissected the mucosa from the right nasal septum close to the vomer.^3,4^ The exact same procedure was performed on the left side. The nasal speculum was applied in each nostril, and the vomer as well as bilateral sphenoid ostium were exposed.

### 2:25 Exposure of the sphenoid sinus

A 4.5-mm diamond drill was used to enter the sphenoid sinus.

### 2:31 Drilling of the clivus

The clivus was drilled with a combination of 4.5- and 3-mm diamond drills. Mucosa of the nasopharynx was opened in a flap fashion with a cautery.

### 2:49 Opening of the nasopharynx mucosa

The middle clivus was rongeured and the dura was exposed. We only opened on the right side considering the location of the tumor.

### 3:41 Opening of the dura

The dura was sharply opened in a flap fashion. Then the arachnoid was opened sharply, and the right sixth nerve was identified. The right hypoglossal nerve was identified inferior to the tumor.

### 4:27 Resection of the tumor capsule

We coagulated the capsule of the tumor with bipolar and the capsule was opened.

### 4:41 Drainage of the tumor cyst

As we aspirated the content of the cyst, gelatinous nodule was encountered and resected.

### 4:54 Resection of the enhanced nodule in MRI

The capsule of the tumor was resected as much as can be safely performed. We inspected inside the tumor, and the brainstem seemed intact. The frozen specimen of the tumor came back as schwannoma; thus, we did not intend complete resection. This is the final view after the resection.

### 6:05 Reconstruction of the dura

A couple of pledgets of Gelfoam was applied, and the fascia lata was inserted in inlay fashion. Another piece of fascia lata was applied in onlay fashion. Abdominal fat was applied, and nasopharynx mucosa was laid back to the original position. Fibrin sealant was sprayed to reduce risk of CSF leak.

### 6:36 Insertion and inflation of Foley catheter

The nasal septal mucosa was returned to the original position, and a 12-Fr Foley balloon was inserted and inflated.

### 6:51 Reconstruction of the nasal septum

The nasal septum was reconstructed with bone fragments, and mucosa was reversed back. Lumbar drainage with one-way pressure control valve system was placed for 72 hours.^5^

### 6:54 Postoperative MRI

Postoperative MRI demonstrated satisfactory subtotal resection of the mass. The patient returned home without any neurological deficits. The final pathology returned as schwannoma, and the patient underwent Gamma Knife surgery for the residual tumor.^6^
